# Risk Assessment of the Increased Occurrence of Congenital Cardiac and Non-Cardiac Defects in Fetuses with a Normal Karyotype after Assisted Fertilization in Comparison to Natural Fertilization Based on Ultrasound Diagnostics

**DOI:** 10.3390/jcm10235630

**Published:** 2021-11-29

**Authors:** Dawid Serafin, Beniamin Oskar Grabarek, Dariusz Boroń, Andrzej Madej, Bartosz Czuba

**Affiliations:** 1 Serafin Clinic, 44-100 Gliwice, Poland; 2 Department of Gynecology and Obstetrics with Gynecologic Oncology, Ludwik Rydygier Memorial Specialized Hospital, 31-826 Kraków, Poland; bgrabarek7@gmail.com (B.O.G.); dariusz@boron.pl (D.B.); 3 Department of Histology, Cytophysiology, and Embryology, Faculty of Medicine, University of Technology, 41-800 Zabrze, Poland; 4 Department of Pharmacology, Faculty of Medicine, University of Technology, 41-800 Zabrze, Poland; andrzejmadej@o2.pl; 5 Women’s Health, Faculty of Health Sciences in Katowice, Medical University of Silesia, 40-752 Katowice, Poland; bczuba64@gmail.com

**Keywords:** assisted reproductive techniques, intrauterine insemination, extracorporeal fertilization, congenital heart defects, congenital non-cardiac defects, prenatal diagnosis

## Abstract

The goal of the study was to assess changes in parameters based on ultrasound examinations—these were Crown Rump Length (CRL), Nuchal Translucency (NT), Fetal Heart Rate (FHR), and Pulsatility Index for Ductus Venosus (DV-PI)—in the first trimester of pregnancy in women in which there was a natural initiation of the pregnancy due to spontaneous ovulation, women in which the pregnancy was initiated as a result of stimulated ovulation, as well as in the group in which pregnancy was achieved through the use of In-Vitro Fertilization (IVF)-assisted reproduction. A total of 1581 women became pregnant without the use of assisted reproduction methods. Out of 283 pregnancies, in 178 patients, induced ovulation was utilized. Next, 137 women had sexual intercourse and became pregnant; 41 of them became pregnant through Intrauterine Insemination (IUI) as a result of Artificial Insemination by Husband (AIH), and 13 became pregnant after Artificial Insemination by Donor (AID). The third group consisted of 105 women subjected to Controlled Ovarian Hyperstimulation (COH). In this group of pregnant women, 53 pregnancies were resultant of Intracytoplasmic Sperm Injection (ICSI), and 52 pregnancies were the result of Intracytoplasmic Morphologically selected Sperm Injection (IMSI). The obtained results did not indicate that the chosen method of fertilization or the chosen ovulation method had a statistically significant effect on the development risk of congenital heart or non-heart defects in the fetus.

## 1. Introduction

Assisted Reproduction Techniques (ART) were introduced in the 1970s, and the term itself covers all methods that assist in becoming pregnant. ART techniques replace the biological functions connected with the process of procreation; thus, they enable conception and pregnancy [1,2]. In Poland, any matter associated with ART is regulated by the Recommendations of the Polish Society of Reproductive Medicine and Embryology (PTMRiE) [2] as well as by the Polish Society of Gynecologists and Obstetricians (PTGP) [3].

Currently, to treat infertility, two methods are used: Artificial Insemination by Husband (AIH) [4] or Artificial Insemination by Donor (AID) [4], alongside In-Vitro Fertilization (IVF) [5].

Intrauterine insemination (artificial insemination) is considered to be an in-vivo method, which means that fertilization happens within the woman’s body. These methods are based on the introduction of male semen or its components into the female reproductive tract after being prepared in a laboratory [6]. The success of the treatment is increased if insemination occurs within the natural cycle or after the stimulation of ovulation. However, it should be considered that according to the recommendations of the PTGP, stimulation is only recommended when there are no more than three pre-ovarian follicles. The highest effectiveness is observed for up to three attempts at intrauterine insemination [7,8].

From the available treatment methods for infertility, no matter the cause, extracorporeal fertilization in in-vitro conditions is characterized by the highest effectiveness. Before carrying out the procedure, an examination of the man’s karyotype should be completed as well as a microdeletion analysis of the AZF region on the Y chromosome. In the case of women, it is recommended that ovarian reserves should be assessed, through which it would be possible to choose the optimal method of ovarian hyperstimulation [2,9,10].

The process of extracorporeal fertilization can be conducted using the classical method based on the introduction, in an in-vitro environment, of prepared sperm to oocytes or, based on the technique, sperm microinjection into the egg, which is known as Intracytoplasmic Sperm Injection (ICSI) or Intracytoplasmic Morphologically selected Sperm Injection (IMSI). This method is recommended with infertility connected with the male factor [1,11]. The choice of the correct ovarian hyperstimulation strategy allows for the growing of many Graafian follicles and the subsequent collection of many mature eggs. Oocytes obtained as a result of a puncture performed under ultrasound control are subjected to a further fertilization process in the conditions of an embryological laboratory. Next, intrauterine transfer from one or two embryos is conducted after the incubation period, which lasts between two and six days [2]. In order to induce ovarian hyperstimulation, drugs belonging to the group of gonadoliberin agonist analogs (short or long protocol) or gonadotropin-releasing hormone (GnRH) are used [2,12,13].

Prenatal diagnosis methods can be split into non-invasive and invasive. In the first group, the following are included: ultrasound examination of the fetus and biochemical examination aimed to mark markers in the blood of pregnant women as well as analysis of fetal cells, which circulate in the mother’s bloodstream. In turn, invasive examination includes trophoblast biopsy, amniocentesis, cordocentesis, and fetoscopy [14,15].

Currently, modern imaging techniques are used in everyday obstetric practice in order to assess the somatic development of the fetus, monitoring the course of the pregnancy, and detecting birth defects in the fetus. It is estimated that the percentage of multiple or large birth defects occurring during pregnancy is much higher than estimated due to the fact that three-fourths of such fetuses undergo a spontaneous abortion. It was also observed that there is a relationship between an increase in the age of the mother and an increase in the occurrence of birth defects, such as neural tube defects, cleft lip and/or palate, congenital inguinal hernia, or heart defects [16,17].

The frequency of heart defects occurring is, in turn, 7.9 for every 10,000 births, and neural tube defects are estimated to be 6.7 for every 10,000 births. In contrast, in the case of small, isolated defects, such as cleft lip and/or palate, which is the most commonly appearing defect of this type of defect, it is determined to appear in approximately 14.9 newborns for every 10,000 live births [16,17]. The main aim of prenatal diagnosis is minimizing adverse obstetric outcomes, which can come as a result of failing to recognize a defect in the fetus; fetus immaturity; or other intrauterine disorders. It was confirmed that together with the age of the mother, the risk of birthing a child burdened with a genetic defect and/or a genetic developmental defect increase. An especially high risk of chromosome trisomy of chromosomes 13, 18, and 21 occurring in fetuses was observed in pregnant women over 35 years of age. It is worth noting, however, that the age of the mother is not the only factor of an increased risk of birth defects in fetuses occurring and also developing [18,19]. Based on the recommendations by PTMRiE and PTGP, ultrasound examination in the first trimester should include a detailed evaluation of the fetal egg, which is an indication of the number of gestational sacs and fetuses in the uterine cavity; assessment of the Fetal Heart Rate (FHR; 110–150 beats/min), Crown Rump Length biometric measurement (CRL—assessment/verification of the gestational age and fetal size), assessment of the fetus anatomy, assessment of the filled fluid space, the space between the subcutaneous tissue, and the skin at the neck level, which is the Nuchal Translucency (NT; at the widest point, markers “from inside to the inside”, horizontal arms of the markers placed on the boundary lines of the NT); as well as the Pulsatility Index for Ductus Venosus (DV-PI)—one of the key diagnostic parameters for congenital malformations [2,3].

Parrazini et al. indicated that fetuses conceived through IVF or ICSI methods, in comparison to those conceived spontaneously, are at an increased risk of developing CHD; in fact, this risk significantly increased in the IVF/ICSI group compared to the spontaneous conception group (pooled OR, 1.45; 95% CI, 1.20–1.76; *p* = 0.0001; I2 = 44%; *p* = 0.08) [20]. Furthermore, Mussa et al. highlighted a 10-fold higher risk of Beckwith–Wiedemann Syndrome occurring in fetuses conceived through the use of ART compared to fetuses that were conceived without the use of ART [21]. Moreover, attention is paid to children born after the use of ART and the altered epigenetic profiles that they possess; these very alterations might play a key role in exploring our understanding of adverse child outcomes after the use of ART [22].

This study aimed to evaluate the risk assessment of the increased occurrence of congenital heart and non-heart defects in fetuses with a normal karyotype after assisted fertilization in comparison to natural fertilization based on the values of CRL, NT, FHR, and DV-PI parameters determined through ultrasound examination in the first trimester of pregnancy.

## 2. Experimental Section

### 2.1. Patient Characteristics

In the assessment performed by the Bioethics Committee of the Medical University of Silesia in Katowice, Poland, the following project is not a medical experiment carried out on people within the meaning of Art. 21 of the Act of 5 December 1996 concerning physician and dentist professions and does not require the approval of the ethics committee. From all of the participants who took part in the study, informed and voluntary consent was obtained from them to participate in this study. Pregnant women were examined in the Department of Obstetrics and Gynecology of the Municipal Hospital in Ruda Śląska (426 cases; 26.94%), the Angelius Provita Medical Center in Katowice (839 cases; 53.07%), and in the Dawimed Dawid Serafin Individual Medical Practice from 2011 to 2016 (316 cases; 19.99%). Assisted reproduction procedures took place in the Angelius Provita Medical Center in Katowice.

In the study, the total number of women was 1581, all of which had a single pregnancy, and in whom the developing fetus had a normal karyotype. Of these, 1298 pregnancies were begun without the use of assisted reproduction methods—82.1% (median age of 34).

In contrast, 283 pregnancies were begun by using assisted reproduction methods (17.9%). From the 283 pregnancies, in 178 patients (52.9%), induced ovulation (IO) was carried out using clomiphene citrate according to a fixed schedule and dose (from the 2nd to the 6th day of the cycle, which is 5 days in total, at a dose of 50 mg a day). Next, after stimulation, 137 women had intercourse naturally and became pregnant—76.7% (median age of 31), and 41 of these women became pregnant after Intrauterine Insemination (IUI) was completed—23.3%, of which 28–68.29% (median age of 32) were the result of completed Artificial Insemination by Husband (AIH), and 13–31.71% (median age of 32) after completed Artificial Insemination by Donor (AID).

The third group of women consisted of 105 women who underwent Controlled Ovarian Hyperstimulation (COH). To achieve the induction of hyperstimulation, follitropin alpha preparations were used at a dose of 225 units daily through subcutaneous injection, gonadotropin—gonal preparation (injections administered subcutaneously once a day for 10 days) as well as cetrotide (injections administered subcutaneously once a day for 5 days from the 7th day of the cycle), using the short protocol with an antagonist—and patients became pregnant using ICSI or IMSI. In the group of pregnant women, 53 pregnancies—50.48% (median age of 33)—were as a result of using the ICSI technique and 52 pregnancies using the IMSI method—49.52%.

Pregnant women with abnormal karyotypes were excluded from the study.

After giving birth, those patients that did not consent to invasive diagnostics were asked to complete a questionnaire during a phone call, and additionally, the medical history of the newborn was analyzed: newborns in which no congenital defects were determined and whose phenotype was assessed as normal were considered to be healthy; newborns with abnormal phenotypes also had an additional assessment of their karyotype, and only those with a normal karyotype were included in the study.

### 2.2. Prenatal Diagnosis Performed in the First Trimester of Pregnancy

Prenatal diagnosis performed in the first trimester of pregnancy included:

Noninvasive ultrasound examination of the fetus (Voluson Expert VE 730, Voluson E6 as well as Voluson E8) carried out between 11 + 0 and 13 + 6 weeks of pregnancy.

During ultrasound examination, the following parameters were assessed:

Crown Rump Length (CRL);

Fetal Heart Rate (FHR);

Nuchal Translucency (NT);

Tricuspid Flow (TR);

Ductus Venosus (DV-PI).

Invasive examination in the case of ultrasound and biochemical diagnostics being determined as abnormal, indicating the possibility of fetal trisomy chromosome pairs 13, 17, and 21 (risk range 1:300) included:

Biopsy of chorionic villus sampling between 11 and 14 weeks of pregnancy;

Diagnostic amniocentesis after 15 weeks of pregnancy.

### 2.3. Statistical Analysis

Statistical analysis of the results, obtained as part of this study, was carried out using the statistical package PQStat version 1.6.4.121 (PQStat Software, Poznań, Poland).

All statistical analyses were carried out using the statistical significance threshold of *p* < 0.05. Firstly, the normality of the distribution of the obtained data was assessed using the Shapiro–Wilk test. The test assumptions were not met, which justified using nonparametric tests for statistical analysis of the results. Therefore, the Mann–Whitney U test was used as a non-parametric equivalent to the Student *t*-test for the two groups. The values of the assessed parameters were presented in the form of a median (Me), lower quartile (Q1), and upper quartile (Q3).

Results of the analyzed quantitative scales in the Congenital Heart Defects (CHD) group and the group without these defects (split into groups depending on the method of ovulation induction and pregnancy after IVF) were compared using the Mann–Whitney U test.

Additionally, the results of the analyzed quantitative scales in the Congenital non-Heart Defect (CnHD) group and the group without these defects (split into groups based on the method of ovulation induction and pregnancy after IVF) were compared using the Mann–Whitney U test. All comparisons were performed between either the group of women with CHD or CnHD and women without either of them.

In turn, the results of the TR and DV scales in the CHD group and the group without these defects as well as in the CnHD group and the group without these defects (split based on the ovulation induction method and pregnancy after IVF) were analyzed using Fisher’s exact test.

## 3. Results

Amongst 1581 pregnant women who underwent prenatal diagnosis in the first trimester of pregnancy, 52 (3.29%) had a congenital defect identified in their fetus, which was confirmed after giving birth. Among the diagnosed congenital defects, 16 (30.77%) were heart defects, and 36 (69.23%) were non-heart defects. In the group of 1529 women (66.71%), both heart defects and non-heart defects were determined. The most commonly occurring heart defect was a Ventricular Septal Defect (VSD). During prenatal diagnosis, six fetuses had Intrauterine Growth Restriction (IUGR) identified, whereas five fetuses were diagnosed with a cleft lip and/or palate as the most common occurring non-heart defect.

Among 178 patients (11.26%) who had induced ovulation and who later became pregnant due to sexual intercourse, three fetuses had a congenital heart defect (1.69%), and in two cases (1.12%), the fetus had a congenital non-heart defect.

In turn, in the group of 1298 women who became pregnant without using the technique of assisted fertilization (82.1%), heart defects were determined in 11 cases (8.47%) and non-heart defects in 32 cases (2.47%).

In contrast, amongst 105 women in whom pregnancy was as a result of extracorporeal in-vitro fertilization (6.64%), congenital heart defects were diagnosed in two cases, one each for the two methods of fertilization (ICSI: 3.77%, IMSI: 3.77%); furthermore, congenital non-heart defects were diagnosed in two cases when fertilization was achieved by a sperm that was not previously morphologically assessed.

### 3.1. Analysis of the Ultrasound Examination of the Fetus with and without Detected Heart Defects among Women Who Became Pregnant without the Use of Assisted Reproduction Methods

Among 1298 women who became pregnant through sexual intercourse without previously induced ovulation, in 11 of these women (0.85%), based on ultrasound examination, the occurrence of a congenital heart defect was determined, which was then additionally confirmed after birth. No heart defect was observed in 32 women (2.47%), and in 1255 cases, neither was observed (96.68%; Table 1).

The first of the assessed parameters that characterize the growth and health of the fetus was the parietal-seat length, which, amongst the fetuses with a diagnosed heart defect, was Me = 66.85 mm (Q1 = 62.25 mm; Q3 = 69.23 mm), whereas amongst fetuses without a determined heart defect, Me = 63 mm (Q1 = 57.7 mm; Q3 = 66.85 mm). Statistical analysis did not indicate any statistically significant differences between the two groups about the CRL (Table 1).

**Figure 1:**
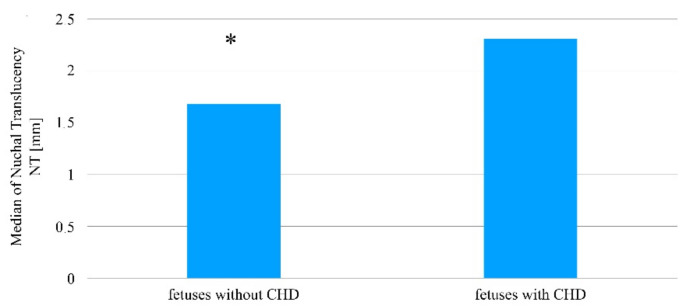
The thickness of the nuchal translucency in fetuses with and without a diagnosed congenital heart defect amongst patients who became pregnant without using assisted reproduction techniques. * Statistically significant difference (Mann–Whitney U test; *p* < 0.05).

In turn, in reference to the thickness of the nuchal translucency, the Mann–Whitney U test indicated the existence of statistically significant differences when comparing the two fetus groups, wherein in the group with diagnosed heart defects, the NT scale results are higher in comparison to the group in which no congenital heart defects was detected (Me = 2.30 mm; Q1 = 1.65 mm; Q3 = 2.68 mm vs. Me = 1.70 mm; Q1 = 1.40 mm; Q3 = 1.98 mm; *p* = 0.0015, Table 1, Figure 1).

**Figure 2:**
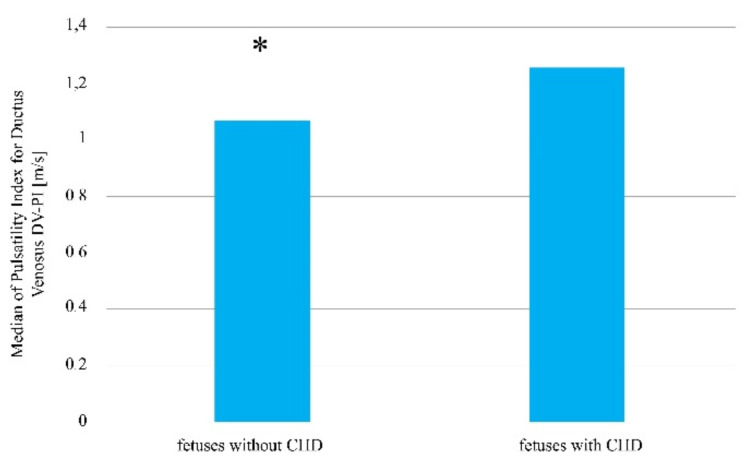
The venous flow rate in fetuses with and without a diagnosed congenital heart defect amongst patients who became pregnant without using assisted reproduction techniques. * Statistically significant difference (Mann–Whitney U test; *p* < 0.05).

In contrast, the FHR values between groups were not statistically significant (*p* > 0.05). In the group of fetuses with heart defects, the FHR value was at the level of Me = 159 beats/minute, Q1 = 157 beats/min, and Q3 = 162.5 beats/min, whereas in the group of fetuses without a diagnosed heart defect, it was Me = 159 beats/min, Q1 = 155 beats/min, and Q3 = 162 beats/min (Table 1). For the DV-PI scale, the following values were observed: in the group with visible heart defects, Me = 1.25 m/s, Q1 = 0.99 m/s, and Q3 = 1.38 m/s; and in the group without a heart defect, Me = 1.06 m/s, Q1 = 0.93 m/s, and Q3 = 1.20 m/s (*p* = 0.007, Table 1, Figure 2).

### 3.2. Analysis of the Ultrasound Examination of the Fetus with or without a Detected Congenital Heart Defect amongst Patients Who Became Pregnant after the Induction of Ovulation Taking into Account the Method of Fertilization

By analyzing the results of the ultrasound examination of 178 pregnant women who underwent induction of ovulation, congenital heart defects could be determined in three fetuses (1.69%) when fertilization was the result of sexual intercourse, while in two cases (1.12%), the fetus had a congenital non-heart defect. In 173 cases—97.19%—neither was observed (Table 2).

**Figure 3:**
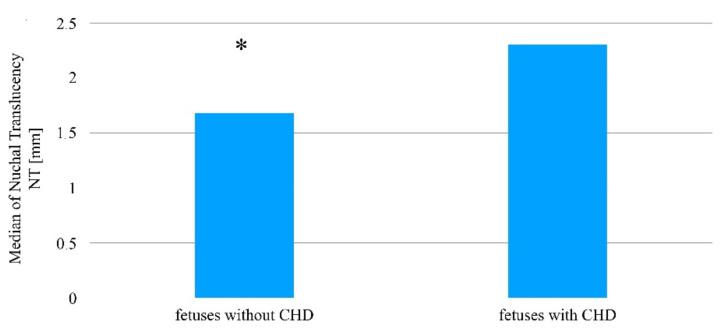
The thickness of nuchal translucency in fetuses with and without a determined heart defect amongst patients after ovulation was induced, who then became pregnant as a result of sexual intercourse. * Statistically significant difference (Mann–Whitney U test; *p* < 0.05).

A statistically significant difference was determined in the case of the nuchal translucency thickness, which was higher in fetuses with congenital heart defects compared to fetuses in which no abnormality was determined (Me = 2.3 mm vs. 1.7 mm; *p* = 0.0007, Figure 3, Table 2).

**Figure 4:**
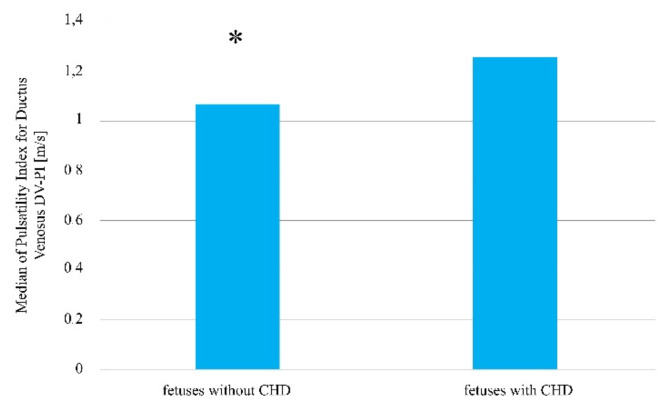
The venous flow rate in fetuses with and without a detected heart defect amongst patients after the induction of ovulation, who later became pregnant as a result of sexual intercourse. * Statistically significant difference (Mann–Whitney U test; *p* < 0.05).

The difference between the venous flow rate in the group of fetuses with determined heart defects in comparison to the group of fetuses without a detected heart defect was also statistically significant (Me = 1.24 m/s vs. 1.05 m/s; *p* = 0.0285, Figure 4, Table 2).

For the remaining parameters assessed in the ultrasound examination, such as CRL or FHR, the Mann–Whitney U test did not indicate any statistically significant differences between groups of fetuses with and without heart defects (Table 3, *p* > 0.05 NS).

In contrast, amongst fetuses started as a result of intrauterine insemination with the semen of husband/partner or donor, the occurrence of congenital heart defects was not observed. Differences in the values of the assessed parameters in the ultrasound examination depending on who the semen donor was were not statistically significant (Table 3, *p* < 0.05).

### 3.3. Analysis of the Ultrasound Examination of the Fetus with or without a Detected Congenital Heart Defect amongst Patients Who Became Pregnant as a Result of In-Vitro Fertilization

Additionally, independent of the technique of intracytoplasmic sperm injection into the oocyte, no statistically significant differences in the values of the parameters assessed in the ultrasound examination were determined. Congenital heart defects were determined in two fetuses (one case after ICSI, one case after IMSI; Table 4, *p* > 0.050.

### 3.4. Analysis of the Ultrasound Examination of the Fetus with a Diagnosed Non-Heart Developmental Defect or after Its Exclusion among Women Who Became Pregnant without Using Assisted Reproduction Techniques

Amongst 1298 women who became pregnant through sexual intercourse without prior induction of ovulation, 32 were diagnosed (2.47%), with the use of ultrasound examination, with a congenital non-heart defect in their fetus, which was later confirmed after birth (Table 5).

**Figure 5:**
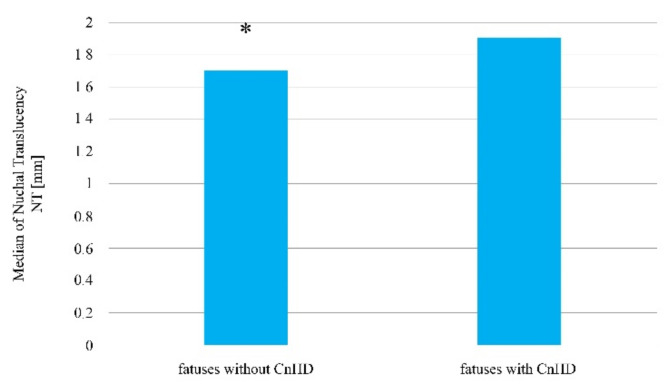
The median nuchal translucency thickness value amongst fetuses with and without a diagnosed congenital non-heart defect amongst patients who became pregnant without the use of assisted reproduction techniques. * Statistically significant difference (Mann–Whitney U test; *p* < 0.05).

The performed statistical analysis indicated that only the nuchal translucency thickness statistically differs in fetuses with developmental non-heart defects in comparison to fetuses in which such defects did not develop (*p* < 0.05, Table 5, Figure 5). In contrast, for indicators describing the parietal-seat length, the fetal heart rate, and blood flow rate through the venous duct, their values did not turn out to be statistically significant (*p* > 0.05, Table 5).

Analysis of the ultrasound examination of the fetus with or without determined congenital non-heart defects amongst patients who became pregnant after the induction of ovulation taking into account the fertilization method.

**Figure 6:**
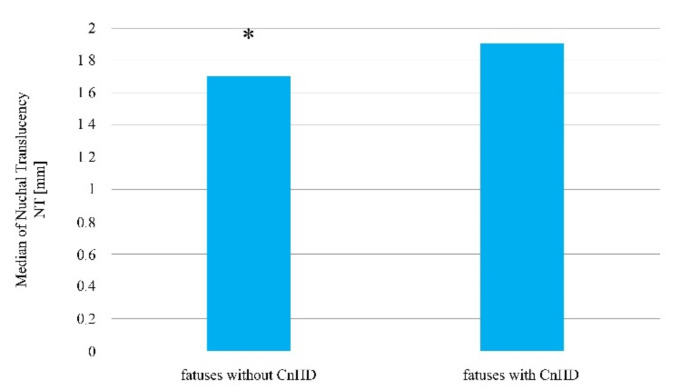
The median nuchal translucency thickness among fetuses with a diagnosed congenital non-heart defect in comparison to fetuses in which such a possibility was excluded, amongst women who became pregnant after the induction of ovulation and sexual intercourse. * Statistically significant difference (Mann–Whitney U test; *p* < 0.05).

By assessing the results of the ultrasound examination of the fetuses with diagnosed congenital non-heart defects (two cases—1.46%, fertilization resulting from sexual intercourse) compared to the fetuses without the burden of a non-heart developmental defect, amongst women who became pregnant after the induction of ovulation and sexual intercourse pregnancies, statistically significant differences were observed in the nuchal translucency thickness (Me = 1.70 mm vs. Me = 1.90 mm; *p* < 0.05, Figure 6). In contrast, for the remaining parameters assessed in the ultrasound examination, the Mann–Whitney U test did not indicate that the differences in the parietal-seat length, the fetal heart rate, and blood flow rate through the venous duct between the two compared groups was statistically significant (*p* > 0.05, Table 6).

Furthermore, the occurrence of congenital non-heart defects in fetuses was analyzed when fertilization occurred as a result of ovulation induction and, afterward, intrauterine insemination with the sperm of the husband/partner or donor. It was determined that this method of fertilization did not induce congenital non-heart malformations.

In Table 7, the differences of values assessed during ultrasound diagnosis in the first trimester of pregnancy, depending on whom the fertilization sperm originated from, are presented.

Statistical analysis did not indicate the occurrence of statistically significant differences in the values of the analyzed parameters (Table 7, *p* > 0.05).

### 3.5. Analysis of the Ultrasound Examination of the Fetus with or without a Detected Congenital Non-Heart Defect among Patients Who Became Pregnant as a Result of Extracorporeal In-Vitro Fertilization

The last stage of ultrasound assessment of the fetus was related to the analysis of routinely conducted determinations during the first trimester of ultrasound in patients who became pregnant using extracorporeal in-vitro fertilization. Non-heart developmental defects were observed only then when fertilization of the egg cell was achieved using sperm, which was not previously assessed morphologically (Table 8).

**Figure 7:**
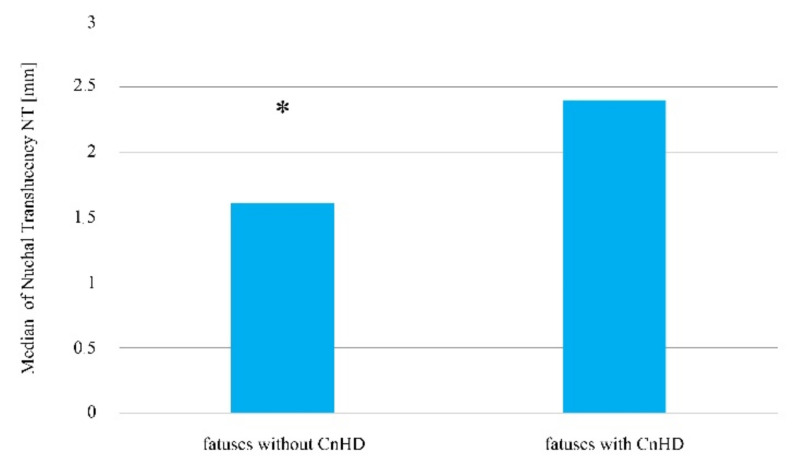
Median nuchal translucency depth amongst fetuses with a diagnosed developmental non-heart defect in comparison to fetuses in whom this possibility was ruled out in women who became pregnant as a result of in-vitro fertilization. * Statistically significant difference (Mann–Whitney U test; *p* < 0.05).

Statistically significant differences in this group of pregnant women were noted only in the case of nuchal translucency depth, which had a higher value in fetuses with non-heart developmental defects in comparison to fetuses without a determined non-heart defect (Table 8, Figure 7, *p* < 0.05).

### 3.6. The Relationship between the Risk of Congenital Heart Defects Occurring in Fetuses Depending on the Used Ovulation Method before Fertilization

Among 178 women who became pregnant as a result of sexual intercourse or through insemination after previous stimulation of ovulation using Clostilbegyt (Clomiphene Citrate), three congenital heart defects were detected in fetuses during prenatal diagnosis; this constitutes to 1.68% of the entirety of group 1. As a result of ovulation stimulation during the ICSI or IMSI fertilization technique, 105 women became pregnant, amongst whom, in two fetuses, a congenital heart defect was identified, which constituted 1.9% of the total population of group 2.

In contrast, in group 3, which is the group with the largest number of women who became pregnant as a result of sexual intercourse without the need for stimulation, 11 fetuses were determined to have a congenital heart defect, which constitutes 0.85% of 1298 women.

Statistical analysis did not confirm that the method in which ovulation is induced affects the risk of congenital heart defects occurring in a fetus (Chi-square = 1.9919, df = 2, *p* = 0.3694; Fisher’s exact test *p* = 0.2074).

### 3.7. The Relationship between the Risk of Congenital Non-Heart Defects Occurring in Fetuses Depending on the Used Ovulation Method before Fertilization

Assessing the occurrence of congenital non-heart defects in the fetuses of the examined women, it can be determined that, in pregnant women in whom ovulation happened spontaneously, these defects occurred in 32 of 1298 fetuses, which forms 2.46% of the entire group.

In turn, in the group of women in whom ovulation was induced and fertilization occurred through the use of the AIH/AID techniques, congenital non-heart defects were determined in two fetuses (1.12%).

The same number of fetuses with non-heart defects was observed in the group of women whose pregnancy was a result of extracorporeal in-vitro fertilization.

Statistical analysis did not indicate that the method of ovulation induction affected the risk of congenital non-heart defects occurring in fetuses (Chi-square= 1.3365, df = 2, *p* = 0.5126; Fisher’s exact test *p* = 0.5921).

### 3.8. The Risk of Congenital Heart Defects Occurring in a Fetus Depending on the Fertilization Technique

By analyzing the risk of congenital heart defects appearing in a fetus depending on the fertilization technique, it can be observed that as a result of AIH or AID fertilization, prenatal diagnosis did not detect the occurrence of a heart defect.

In turn, when fertilization took place through extracorporeal in-vitro fertilization, one fetus was found with a developmental defect regardless of whether the sperm used for fertilization had been previously assessed in terms of morphology.

However, from 1435 fetuses, if the fertilization was as a result of sexual intercourse, congenital heart defects were detected in 14 fetuses.

Statistical analysis did not indicate that the occurrence of heart defects in fetuses depended on the fertilization technique (Chi-square= 1.2739, df = 4, *p* = 0.8658; Fisher’s exact test *p* = 0.5404).

### 3.9. The Relationship between the Risk of Congenital Non-Heart Defects Occurring in a Fetus Depending on the Fertilization Technique

In this study, the potential relationship between the occurrence of congenital non-heart defects in fetuses depending on the utilized fertilization technique was also assessed.

Non-heart defects were diagnosed in only those fetuses conceived as a result of sexual intercourse, 34 of 1435 cases, as well as ICSI conceptions, 2 of 53 cases.

Developing fetuses as a result of using the remaining techniques did not have congenital non-heart defects.

Statistical analysis did not indicate that the occurrence of congenital non-heart defects in fetuses depended on the fertilization technique (Chi-square= 2.7554, df = 4, *p* = 0.5996; Fisher’s exact test *p* = 0.6841).

## 4. Discussion

In accordance with the recommendations of PTMRiE and PTGP, prenatal diagnosis includes the performance of ultrasound examination, and the specificity of the test is different at each stage of pregnancy development. The fundamental goal of ultrasound examination of the pregnancy is minimizing adverse obstetric outcomes, which can come as a result of failing to recognize a defect in the fetus, fetus immaturity, or other intrauterine disorders. Currently, no assessments exist that may suggest that ultrasound examination negatively affects the development of the fetus. The aim of the ultrasound examination in the first trimester of the pregnancy is the assessment of the structure of the egg and the risk of the most common chromosomal aberrations occurring.

First, the possibility of a relationship existing between using assisted reproduction techniques and the occurrence of congenital heart defects was assessed, as such defects form a significant troublesome disease that burdens the developing fetus [23,24]; for instance, the most commonly diagnosed heart defect during ultrasound examination was a defect of the interventricular septum.

According to available epidemiological data, the percentage of detected heart defects during conducted prenatal diagnosis is on a relatively low level; furthermore, this situation occurs even in developed countries and ranges from 30% to 60% [25,26].

For example, in the Netherlands, despite the existence of an early prenatal diagnosis program, the rate of diagnosed heart defects in fetuses was 45.1%. As the authors point out, the main cause of such a low detectability of CHD could be a lack of ultrasound analysis skills in the context of congenital defects as well as the occurrence of obesity or polyhydramnios in pregnant women, which makes diagnosis significantly harder [27,28,29].

It can be observed that neck translucency thickness (NT) as well as the blood flow rate through the venous line (DV-PI) were statistically significantly higher in fetuses burdened with a heart defect in comparison to fetuses in which no such defects were determined when fertilization occurred as a result of sexual intercourse in the group of women in which no assisted reproduction techniques were used (11 of 1298 cases) as well as in the group of women after pharmacologically induced ovulation (3 of 137 cases). However, the obtained results did not indicate that any fetus which developed as a result of intracytoplasmic injection was fraught with a congenital heart defect; therefore, the likely cause of a heart defect not being identified amongst these fetuses may result from the fact that the sperm used for fertilization is selected before it is introduced to the egg [30].

Likewise, when fertilization occurred in in-vitro conditions, two cases were noted of fetuses with a diagnosed congenital heart defect independent of whether the semen chosen for fertilization was randomly selected or selected morphologically. However, in this group of pregnant women, ultrasound diagnosis did not indicate that the value of any of the assessed parameters differed significantly between fetuses with heart defects compared to fetuses without this developmental anomaly. One of the possible causes as to why there was no statistical significance determined could be the relatively small size of the group subjected to in-vitro fertilization and the percentage of detected defects; despite this, it should also be considered that the IVF procedure is cost intensive, the rules for its implementation are strict, and additionally, many potential pairs who could possibly use such treatment decide not to, mainly due to discouragement from the lack of expected results from previous treatment. Consequently, these factors significantly limit the size of the group.

Similar observations of the influence of using ART on the frequency of the occurrence of congenital heart defects noted in this work were also described by Iwashima et al. [28], who, in their study, supervised a total of 2716 pregnant women.

Of this group, 2317 women became pregnant without the use of ART, whereas in 399, ART was used, of which 142 patients used pharmacological treatment aiming to induce ovulation; in 56 women, AIH was performed, and in 159 women, the pregnancy was a result of IVF, of which 42 women underwent the ICSI procedure. Differential diagnosis was based on electrocardiographic examination of the fetus or newborn. Among the fetuses resulting from assisted reproduction techniques, in 11, a congenital heart defect was diagnosed in comparison to 94 confirmed cases when ART was not used. In summary, Iwashima et al. [31] determined there was no statistically significant relationship between the use of assisted reproductive techniques and the risk of developing congenital heart defects in fetuses [32], which is consistent with the observations made in this study.

In turn, Wen et al. [33] carried out a cohort examination on a group of 507,390 women, assessing the risk of development of congenital heart defects in fetuses after fertilization using IVF.

These researchers did not observe the existence of a relationship between in-vitro fertilization and fertilization as a result of sexual intercourse without the use of assisted reproduction techniques. Alternatively, they noted that when determining the risk of development of developmental anomalies, more than one risk factor should be taken into account simultaneously [34].

Furthermore, a heart defect was also diagnosed in fetuses amongst women whose ovulation was induced, however, when fertilization came as a result of sexual intercourse, i.e., without the need to perform intrauterine insemination with the sperm of the husband/partner or donor. Considering reports from other centers that assessed the influence of drugs used for hyperstimulation of the ovaries on the health of the fetus, including the development of congenital defects, it can be determined that the used ovulation induction technique is not significantly connected with an increase in the risk of developmental anomalies occurring during fetal life [35,36].

Nonetheless, however, based on the performed ultrasound examination in the first trimester of pregnancy, it can be assumed that nuchal translucency thickness and venous duct flow rate constitute significant markers based on which fetuses with congenital heart defects can be determined from fetuses not burdened by such a malformation when the pregnancy is a result of in vivo fertilization. Therefore, such a conclusion seems justified as for those two parameters—NT and DV-PI—statistical analysis indicated the occurrence of statistically significant differences in the compared groups. Additionally, based on the current, available literature, the relationship between the NT value and the risk of congenital heart defects occurring has been documented [37,38].

Alanen et al. [38], over the course of three years, analyzed the relationship between the NT value and the risk of CHD developing. As part of this study, 31,114 pregnant volunteers were included, in which a screening ultrasound examination was carried out in the first trimester of pregnancy. Assuming the cut-off point for the NT value at NT > 3.5 cm, 79 fetuses were determined to have heart defects, of which 21.5% were noted to have the occurrence of chromosomal aberrations. Importantly, it is also worth noting that in the study described by Alen et al. [38], ultrasound was performed by more than one doctor; furthermore, in order to perform ultrasound screening of the fetus, it was not necessary to obtain additional certificates [38].

In turn, in the case of this study, all ultrasound examinations were performed personally by myself (D.S.), using one ultrasound machine; therefore, the risk of an incorrect NT value reading while also obtaining false positive or negative results was minimized.

The ultrasound apparatus in obstetric, and gynecological diagnostics should have real-time 2D presentation; at least 128-degree grayscale; and it should be able to measure distances (at least two measurements), circumference, and surface area as well as the obstetric program. Furthermore, the apparatus should also be equipped with transabdominal and transvaginal heads with the possibility of photographic and electronic documentation. Additionally, a valuable addition to the functions of the ultrasound of the machine is the Doppler color option. Therefore, the accuracy and reliability of the results depend on the apparatus utilized during ultrasound examination. It is also worth remembering that even the most modern ultrasound apparatus is not enough for a thorough and effective examination if the presence of an experienced specialist who can accurately interpret the image on the monitor is missing. The experience of the person carrying out the examination should be supported with a Certificate of Competence given by The Fetal Medicine Foundation (FMF). Therefore, the combination of professional equipment with the capabilities of a doctor guarantees the accuracy and reliability of the resulting measurements [1,2,3].

Furthermore, it should also be considered that in the examination carried out by Alanen et al. [35], the value NT > 3.5 cm constituted the cut-off point, while in the present study, in the ultrasound examination, the NT value did not exceed 2.5 cm and was higher in CHD fetuses. Moreover, the authors themselves admit that when the NT cut-off point was lowered to 2.0 and 1.5 cm, respectively, the heart defect detection rate increased to 25.3% and 46.8%, respectively [38].

Swelling of the nape of the neck as a result of congenital heart defects (increased nuchal translucency value) could most likely be connected with disturbed development of the lymphatic system [39,40] and is strongly correlated with congenital heart defects [41,42]. Furthermore, alongside an increase in NT value, the risk of CHD developing increases [43]; additionally, the currently used analyses between NT and CHD concentrate on learning about and understanding the molecular mechanisms related to disturbed embryonic development of the lymphatic and circulatory systems [44].

The second significant indicator connected with a heightened risk of congenital heart defects occurring in a fetus is the DV-PI parameter, determining the speed of blood flow in the venous tract; in particular, in fetuses with CHD, a significantly faster flow of blood in the venous tract was observed in comparison to fetuses not burdened with CHD.

The Ductus Venosus (DV) significantly regulates the supply of blood nutrients from the umbilical vein between the liver and heart [45]; therefore, the flow rate through the venous duct is indicated to have a potential use in the forecasting, among others, the risk of miscarriage, stillbirth, low birth weight, abnormal fetal growth, and the development of a serious congenital heart defect [46].

In this study, in the case of fetuses with a heart defect, the venous flow rate through the DV was approximately 1.25 m/s, whereas in healthy fetuses, the DV-PI was at a level no higher than 1.0 m/s. Similar observations were noted by Baran et al. [47], who reported that fetuses with developmental anomalies had an average DV-PI value of 1.22 m/s [47]. Additionally, in one of the cohort examinations regarding DV-PI reference values, it was indicated to interpret venous flow rates below 0.93 m/s and above 1.22 m/s as abnormal [48], which is compatible with our observations in this study. Likewise, the inclusion of the DV-PI parameter during ultrasound examination is a significant factor that increases the rate of heart defect diagnosis in a fetus [49].

As part of this study, the influence of the fertilization method utilized when using assisted reproduction techniques on non-heart defects (CnHD) occurring in fetuses was also assessed. The largest number of fetuses with CnHD was noted when fertilization was achieved as a result of sexual intercourse, of which, in 32 out of 34 cases, ART was not used. In turn, in the group of patients who underwent in-vitro fertilization, one case of CnHD was noted after the ICSI procedure.

By analyzing the obtained data in the context of developmental non-heart defects, only one statistically significant difference amongst the assessed parameters was observed. Consequently, amongst the fetuses in which congenital non-heart defects were observed and confirmed, the NT value was higher than in fetuses without this type of abnormality.

Therefore, it is indicated that in the cases where a normal karyotype is determined, a heightened NT, especially above 3.5 mm, is connected with the risk of developing a serious structural abnormality, particularly connected with the cardiovascular, digestive, and musculoskeletal systems [50,51]. Baer et al. [52] analyzed the relationship between a higher NT value at ≥3.5 mm or ≥95th percentile for CRL with non-heart anomalies. Moreover, babies that, in the prenatal examination, had a higher NT value were at risk of having one serious non-heart structural defect (any defect, RR: 1.6; 95% CI: 0.3–1.9; many defects, RR: 2.1; 95% CI: 1.3–3.4) [52].

In this study, the most common non-heart developmental defect was a cleft lip and/or palate, which is found in Poland at a frequency of 2–3 per 1000 births [53]. For this defect to occur, genetic factors have an influence, including the abnormal expression of genes included in the growth factor family and their receptors, which consequently is connected with the occurrence of abnormal transduction of signals along signaling pathways [54]. Furthermore, this is also pointed to the fact that the risk of CnHD developing is higher when the mother is not supplemented with folic acid in pregnancy [55]; moreover, the importance of environmental factors is also highlighted, including the age of the parents-to-be. Interestingly, in the situation where the father is over 40 years of age, the risk of a cleft lip and/or palate occurring is 58%, whereas in mothers who are over 40 years of age, this risk is 28% [56].

Of course, one should bear in mind the extremely dynamic development of non-invasive prenatal testing (NIPT), also known as non-invasive prenatal screening (NIPS), based, inter alia, on cell-free fetal DNA (cfDNA) analysis in the maternal bloodstream. These are not yet recognized diagnostic methods, but in light of the emerging ethical dilemmas for invasive prenatal testing, NIPT is gaining importance. It is a highly sensitive, specific screening technique that can be used at an early stage, e.g., detection of chromosomal aneuploidies or submicroscopic copy number variations (CNVs) [57,58]. Zaami et al. [58] also emphasized that the development of non-invasive methods is an important step in counteracting dangerous and unethical trends in selective reproductive technologies (SRTs) and eugenics [58].

## 5. Conclusions

In summary, based on the assessed parameters in the ultrasound examination of the fetus, a higher risk of congenital heart and non-heart defects in fetuses with a normal karyotype after assisted reproduction was not observed in comparison to natural insemination. Furthermore, the presented analysis confirmed the validity of the prenatal diagnosis, which included ultrasound assessment of the fetus, especially the assessment of the thickness of the nuchal translucency and the flow rate through the venous duct in the early detection of congenital heart and non-heart defects in fetuses with a normal karyotype.

## Figures and Tables

**Table 1:** Assessment of the parameters describing the growth and health of the fetus with or without detected heart defects, based on ultrasound examination in a group of women who became pregnant without using assisted reproduction techniques.

Parameter	Fetus Group	Q1	Me	Q3	*p* < 0.05 (Mann–Whitney U Test)
CRL (mm)	No heart defect (1255 cases; 96.98%)	57.70	63.00	68.80	*p* > 0.05 NS
	Heart defect (11 cases; 0.85%)	62.25	66.85	69.23	
NT (mm)	No heart defect (1255 cases; 96.98%)	1.40	1.70	1.98	*p* = 0.0015
	Heart defect (11 cases; 0.85%)	1.65	2.30	2.68	
FHR (beats/min)	No heart defect (1255 cases; 96.98%)	155.00	159.00	162.00	*p* > 0.05 NS
	Heart defect (11 cases; 0.85%%)	157.00	159.00	162.50	
DV-PI (m/s)	No heart defect (1255 cases; 96.98%)	0.93	1.06	1.20	*p* = 0.0068
	Heart defect (11 cases; 0.85%)	0.99	1.25	1.38	

CRL, Crown Rump Length; NT, Nuchal Translucency; FHR, Fetal Heart Rate; DV-PI, Pulsatility Index for Ductus Venosus.

**Table 2:** Assessment of the parameters describing the growth and health of the fetus with or without a congenital heart defect based on ultrasound examination in a group of women who became pregnant as a result of sexual intercourse after the induction of ovulation.

Parameter	Fetus Group	Q1	Me	Q3	*p* < 0.05 (Mann–Whitney U Test)
CRL (mm)	Heart defect (3 cases; 1.69%)	57.80	63.20	69.00	*p* > 0.05 NS
	No heart defect (173 cases; 97.19%)	62.50	66.80	69.15	
NT (mm)	Heart defect (3 cases; 1.69%)	1.50	1.70	2.00	*p* = 0.0007
	No heart defect (173 cases; 97.19%)	1.95	2.30	3.00	
FHR (beats/min)	Heart defect (3 cases; 1.69%)	155.00	159.00	162.00	*p* > 0.05 NS
	No heart defect (173 cases; 97.19%)	157.00	158.00	162.00	
DV-PI (m/s)	Heart defect (3 cases; 1.69%)	0.92	1.05	1.20	*p* = 0.0285
	No heart defect (173 cases; 97.19%)	0.97	1.24	1.30	

CRL, Crown Rump Length; NT, Nuchal Translucency; FHR, Fetal Heart Rate; DV-PI, Pulsatility Index for Ductus Venosus.

**Table 3:** Assessment of the parameters describing the growth and health of the fetus without a congenital heart defect based on ultrasound examination in a group of women who became pregnant as a result of Artificial insemination by Husband or Donor.

Parameter	Way of Fertilization (41 Cases; 100%)	Q1	Me	Q3	*p* < 0.05 (Mann–Whitney U Test)
CRL (mm)	AIH (28 cases; 68.29%)	52.90	58.50	64.03	*p* > 0.05 NS
	AID (13 cases; 31.72%)	58.90	60.60	65.00	
NT (mm)	AIH (28 cases; 68.29%)	1.30	1.60	1.93	*p* > 0.05 NS
	AID (13 cases; 31.72%)	1.50	1.70	2.00	
FHR (beats/min)	AIH (28 cases; 68.29%)	158.75	161.00	164.00	*p* > 0.05 NS
	AID (13 cases; 31.72%)	155.00	161.00	170.00	
DV-PI (m/s)	AIH (28 cases; 68.29%)	1.00	1.08	1.15	*p* > 0.05 NS
	AID (13 cases; 31.72%)	1.00	1.11	1.21	

CRL, Crown Rump Length; NT, Nuchal Translucency; FHR, Fetal Heart Rate; DV-PI, Pulsatility Index for Ductus Venosus.

**Table 4:** Assessment of the parameters describing the growth and health of the fetus without a congenital heart defect based on ultrasound examination in a group of women who became pregnant as a result of the Intracytoplasmic Sperm Injection technique or the Injection of Morphologically Selected Sperm.

Parameter	Way of Fertilization (105 Cases; 100%)	Q1	Me	Q3	*p* < 0.05 (Mann–Whitney U Test)
CRL (mm)	ICSI (53 cases; 50.48%)	55.80	61.10	66.90	*p* > 0.05 NS
	IMSI (52 cases; 49.52%)	60.85	66.20	73.60	
NT (mm)	ICSI (53 cases; 50.48%)	1.30	1.60	1.90	*p* > 0.05 NS
	IMSI (52 cases; 49.52%)	1.45	1.70	1.90	
FHR (beats/min)	ICSI (53 cases; 50.48%)	156.00	161.00	164.25	*p* > 0.05 NS
	IMSI (52 cases; 49.52%)	157.00	161.00	163.50	
DV-PI (m/s)	ICSI (53 cases; 50.48%)	0.93	1.00	1.13	*p* > 0.05 NS
	IMSI (52 cases; 49.52%)	1.00	1.16	1.26	

CRL, Crown Rump Length; NT, Nuchal Translucency; FHR, Fetal Heart Rate; DV-PI, Pulsatility Index for Ductus Venosus; ICSI, Intracytoplasmic Sperm Injection; IMSI, Intracytoplasmic morphologically selected sperm injection.

**Table 5:** Assessment of the parameters describing the growth and health of the fetus based on ultrasound examination in a group of women who became pregnant without the use of assisted reproduction techniques.

Parameter	Fetus Group	Q1	Me	Q3	*p* < 0.05 (Mann–Whitney U Test)
CRL (mm)	No congenital non-heart defect (1255 cases; 96.98%)	57.80	63.00	68.80	*p* > 0.05 NS
	congenital non-heart defect (32 cases; 2.47%)	56.47	62.80	70.00	
NT (mm)	No congenital non-heart defect (1255 cases; 96.98%)	1.40	1.70	1.92	*p* = 0.0207
	congenital non-heart defect (32 cases; 2.47%)	1.50	1.90	2.93	
FHR (beats/min)	No congenital non-heart defect (1255 cases; 96.98%)	155.00	159.00	162.00	*p* > 0.05 NS
	congenital non-heart defect (32 cases; 2.47%)	152.75	159.50	166.00	
DV-PI (m/s)	No congenital non-heart defect (1255 cases; 96.98%)	0.93	1.06	1.20	*p* > 0.05 NS
	congenital non-heart defect (32 cases; 2.47%)	0.98	1.10	1.17	

CRL, Crown Rump Length; NT, Nuchal Translucency; FHR, Fetal Heart Rate; DV-PI, Pulsatility Index for Ductus Venosus.

**Table 6:** Assessment of parameters describing the growth and health of the fetus with determined non-heart developmental defects or after the exclusion of their possibility based on ultrasound examination in the group of women who became pregnant after the induction of ovulation as a result of sexual intercourse.

Parameter	Fetus Group (137 Cases; 100%)	Q1	Me	Q3	*p* < 0.05 (Mann–Whitney U Test)
CRL (mm)	No congenital non-heart defect (135 cases; 98.54%)	57.90	63.30	69.00	*p* > 0.05 NS
	congenital non-heart defect (2 cases; 1.46%)	56.15	61.35	69.32	
NT (mm)	No congenital non-heart defect (135 cases; 98.54%)	1.50	1.70	2.00	*p* = 0.0262
	congenital non-heart defect (2 cases; 1.46%)	1.50	1.90	3.08	
FHR (beats/minute)	No congenital non-heart defect (135 cases; 98.54%)	155.00	159.00	162.00	*p* > 0.05 NS
	congenital non-heart defect (2 cases; 1.46%)	155.00	160.00	166.25	
DV-PI (m/s)	No congenital non-heart defect (135 cases; 98.54%)	0.92	1.05	1.20	*p* > 0.05 NS
	congenital non-heart defect (2 cases; 1.46%)	0.99	1.10	1.16	

CRL, Crown Rump Length; NT, Nuchal Translucency; FHR, Fetal Heart Rate; DV-PI, Pulsatility Index for Ductus Venosus; ICSI, Intracytoplasmic Sperm Injection; IM-SI, Intracytoplasmic morphologically selected sperm injection.

**Table 7:** Assessment of parameters describing the growth and health of fetuses based on ultrasound examination in a group of women who became pregnant after the induction of ovulation as a result of intrauterine insemination with the sperm of the husband/partner or donor.

Parameter	Way of Fertilization	Q1	Me	Q3	*p* < 0.05 (Mann–Whitney U Test)
CRL (mm)	AIH (28 cases; 68.29%)	52.90	52.90	52.90	*p* > 0.05 NS
	AID (13 cases; 31.72%)	58.50	58.50	58.50	
NT (mm)	AIH (28 cases; 68.29%)	1.30	1.60	1.93	*p* > 0.05 NS
	AID (13 cases; 31.72%)	1.50	1.70	2.00	
FHR (beats/min)	AIH (28 cases; 68.29%)	158.75	161.00	164.00	*p* > 0.05 NS
	AID (13 cases; 31.72%)	155.00	161.00	170.00	
DV-PI (m/s)	AIH (28 cases; 68.29%)	1.00	1.08	1.15	*p* > 0.05 NS
	AID (13 cases; 31.72%)	1.00	1.11	1.21	

CRL, Crown Rump Length; NT, Nuchal Translucency; FHR, Fetal Heart Rate; DV-PI, Pulsatility Index for Ductus Venosus; AIH, Artificial Insemination by Husband; AID, Artificial Insemination by Donor.

**Table 8:** Assessment of the parameters describing the growth and health of the fetus with or without a congenital non-heart defect based on ultrasound examination in a group of women who became pregnant as a result of the intracytoplasmic sperm injection technique or the injection of morphologically selected sperm.

Parameter	Intracytoplasmic Sperm Injection Technique		Q1	Me	Q3	*p* < 0.05 (Mann–Whitney U Test)
CRL (mm)	ICSI (53 cases; 50.48%)	No defect (96.23%)	55.80	60.80	66.25	*p* > 0.05 NS
		Defect (2 cases; 3.77%)	66.90	67.00	67.10	
	IMSI (52 cases; 49.52%) No defect		61.13	67.45	74.25	
NT (mm)	ICSI (53 cases; 50.48%)	No defect (96.23%)	1.30	1.60	1.80	*p* = 0.0445
		Defect (2 cases; 3.77%)	2.20	2.40	2.60	
	IMSI (52 cases; 49.52%) No defect		1.48	1.70	1.90	
FHR (beats/min)	ICSI (53 cases; 50.48%)	No defect (96.23%)	156.00	161.00	164.50	*p* > 0.05 NS
		Defect (2 cases; 3.77%)	163.25	163.50	163.75	
	IMSI (52 cases; 49.52%) No defect		157.00	161.00	164.00	
DV-PI (m/s)	ICSI (53 cases; 50.48%)	No defect (96.23%)	0.93	1.00	1.14	*p* > 0.05 NS
		Defect (2 cases; 3.77%)	0.82	0.82	0.83	
	IMSI (52 cases; 49.52%) No defect		1.00	1.16	1.27	

CRL, Crown Rump Length; NT, Nuchal Translucency; FHR, Fetal Heart Rate; DV-PI, Pulsatility Index for Ductus Venosus; ICSI, Intracytoplasmic Sperm Injection; IMSI, Intracytoplasmic Morphologically selected Sperm Injection.

## Data Availability

The data used to support the findings of this study are included in the article. The data will not be shared due to third-party rights and commercial confidentiality.
